# 2-Chloro-5-fluoro-6-methyl-*N*-*o*-tolyl­pyrimidin-4-amine

**DOI:** 10.1107/S160053681300812X

**Published:** 2013-04-05

**Authors:** Yufei Jiang, Kong Wu, Dongmei Cui, Wei Zhou

**Affiliations:** a College of Pharmaceutical Science, Zhejiang University of Technology, Hangzhou 310032, People’s Republic of China

## Abstract

In the title compound, C_12_H_11_ClFN_3_, the benzene ring forms a dihedral angle of 72.43 (5)° with the pyrimidine ring. In the crystal, N—H⋯N hydrogen bonds link the mol­ecules into a chain running along the *c* axis.

## Related literature

For background to and applications of fluoro-pyrimidines, see: Riccaboni *et al.* (2010 ▶). For the anti­tumor activity of 4-aniline-substituted 5-fluoro­pyrimidines, see: Lawrence *et al.* (2012 ▶).
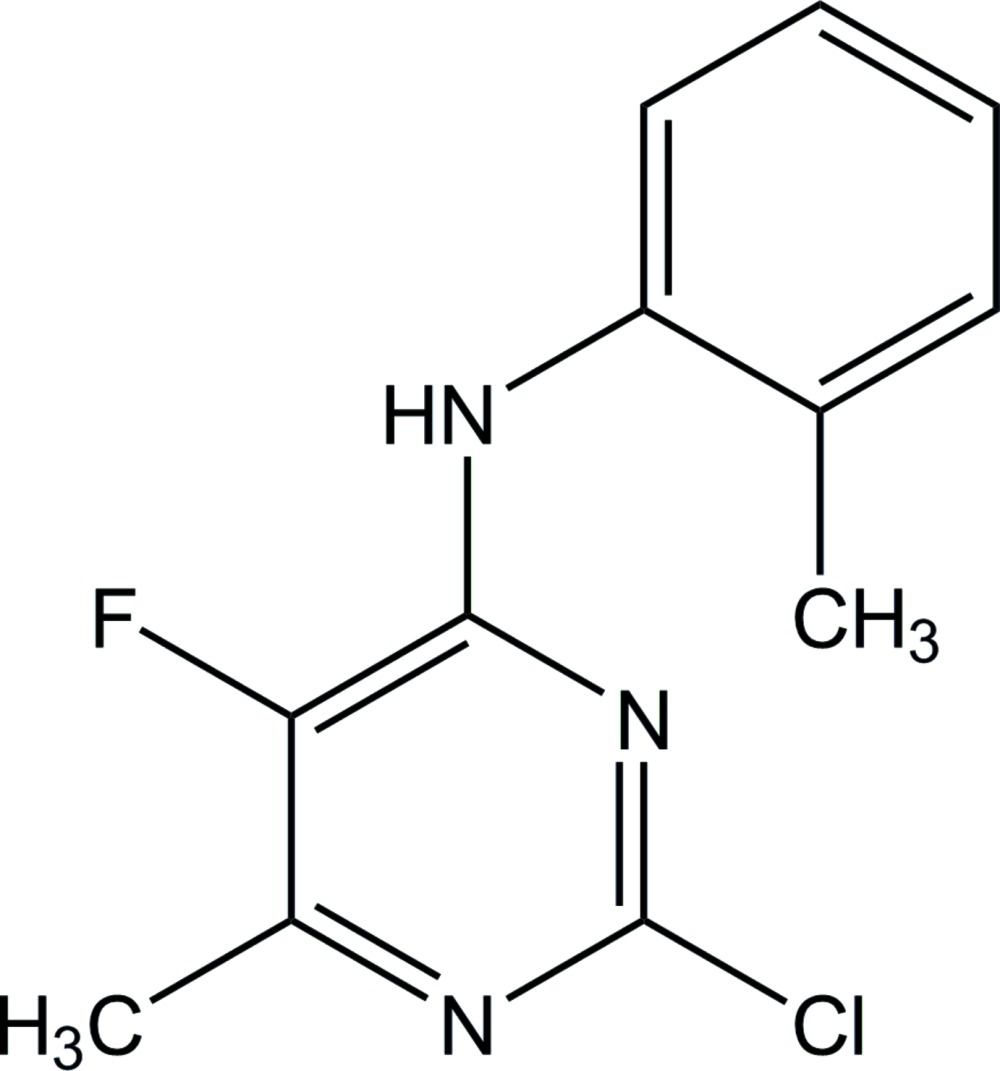


## Experimental

### 

#### Crystal data

C_12_H_11_ClFN_3_
*M* *_r_* = 251.69
Monoclinic, 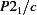

*a* = 12.0593 (7) Å
*b* = 8.3684 (4) Å
*c* = 12.8611 (6) Å
β = 113.021 (6)°
*V* = 1194.54 (11) Å^3^
*Z* = 4
Mo *K*α radiation
μ = 0.31 mm^−1^
*T* = 293 K
0.5 × 0.3 × 0.2 mm

#### Data collection

Enraf–Nonius CAD-4 diffractometer
4692 measured reflections
2125 independent reflections
1750 reflections with *I* > 2σ(*I*)
*R*_int_ = 0.014
3 standard reflections every 60 min intensity decay: none

#### Refinement

*R*[*F* ^2^ > 2σ(*F* ^2^)] = 0.034
*wR*(*F* ^2^) = 0.096
*S* = 1.04
2125 reflections
156 parameters
H-atom parameters constrained
Δρ_max_ = 0.17 e Å^−3^
Δρ_min_ = −0.21 e Å^−3^

### 

Data collection: *CAD-4 Software* (Enraf–Nonius, 1994 ▶); cell refinement: *CAD-4 Software* (Enraf–Nonius, 1994 ▶); data reduction: *XCAD4* (Harms & Wocadlo, 1995 ▶); program(s) used to solve structure: *SHELXS97* (Sheldrick, 2008 ▶); program(s) used to refine structure: *SHELXL97* (Sheldrick, 2008 ▶); molecular graphics: *ORTEP-3 for Windows* (Farrugia, 2012 ▶); software used to prepare material for publication: *SHELXL97*.

## Supplementary Material

Crystal structure: contains datablock(s) global, I. DOI: 10.1107/S160053681300812X/is5256sup1.cif

Structure factors: contains datablock(s) I. DOI: 10.1107/S160053681300812X/is5256Isup2.hkl

Supplementary material file. DOI: 10.1107/S160053681300812X/is5256Isup3.cml

Additional supplementary materials:  crystallographic information; 3D view; checkCIF report

## Figures and Tables

**Table 1:** Hydrogen-bond geometry (Å, °)

*D*—H⋯*A*	*D*—H	H⋯*A*	*D*⋯*A*	*D*—H⋯*A*
N1—H1⋯N2^i^	0.86	2.34	3.0768 (19)	145

Symmetry code: (i) 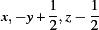.

**Fig. 1.**
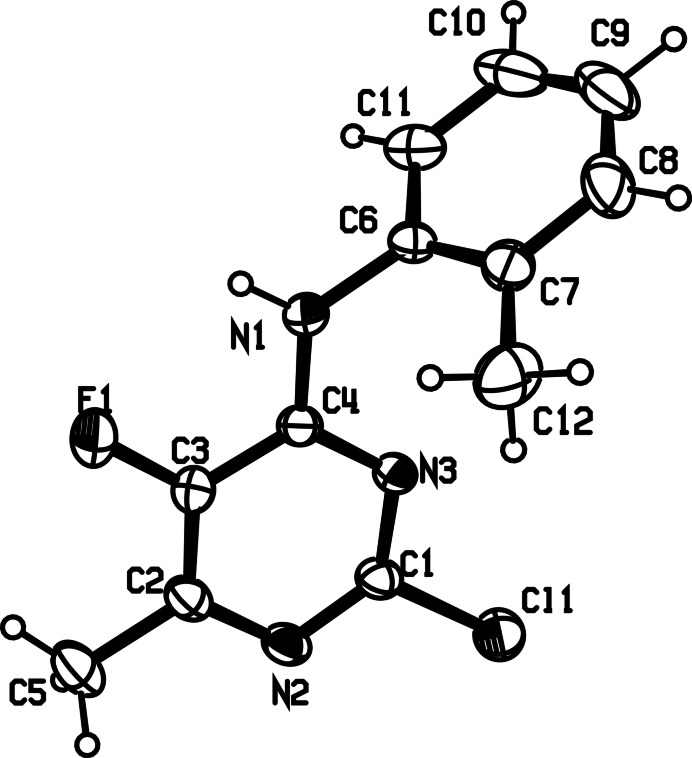
The molecular structure of the title compound with 30% probability displacement ellipsoids.

**Fig. 2.**
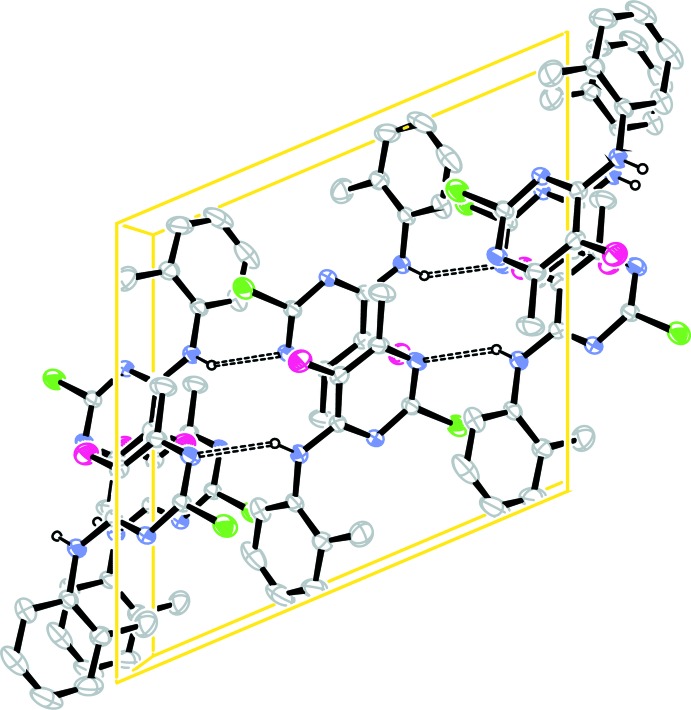
Packing diagram of the title compound, viewed normal to (010), showing hydrogen bonds (dashed lines). For clarity, H atoms not involved in the hydrogen bonds have been omitted.

## supplementary crystallographic information

### Comment

The fluoro-containning pyrimidine skeleton was found in many biologically active molecules (Riccaboni *et al.*, 2010). Especially, the 4-anilines-substituted derivatives of 5-fluoropyrimidine were proved possessing obvious antitumor activity in recent years (Lawrence *et al.*, 2012). Owing to our interest in this area, we have prepared a series of 5-fluoropyrimidine derivatives substituted with anilines. In a continuation of our SAR investigations, we present here the crystal structure of the title compound, (1).

In (1) (Fig. 1), the atoms N1/H1 and C6 are co-planar well with the pyrimidine ring [r.m.s. 0.007 (1) Å], indicating a good conjugate system between the atom N1 and pyrimidine. The plane of *o*-toluidine moiety is torsional toward the pyrimidine ring, showing a dihedral angle of 72.43 (5)°. The amino group is involved in the formation of intermolecular N—H···N hydrogen bond (Table 1). In the crystal (Fig. 2), the intermolecular N—H···N hydrogen bonds link the molecules into one-dimensional chains running along the *c* axis.

### Experimental

In a tube-reactor was added a mixture of 2,4-dichloro-5-fluoro-6-methylpyrimidine (0.181 g, 1.0 mmol), *o*-toluidine (0.107 g, 1.0 mmol), KHCO_3_ (0.1 g, 1.0 mmol) and 1.0 ml DMSO. The mixture was heated at 333 K until the TLC test showed that the reaction is complete. Then the mixture was diluted with 30 ml e thyl acetate, washed with 30 ml water for three times, dried with anhydrous sodium sulfate, and concentrated under reduced pressure. The crude product was purified by column chromatography (petroleum ether / ethyl acetate = 8/1) to give a white solid (0.238 g, yield 94.5%, m.p. 430–432 K). Since the crystal product was not found to be suitable for X-ray diffraction studies, a few solids were dissolved in ethyl acetate, which was allowed to evaporate slowly to give colourless crystals of (1) suitable for X-ray diffraction studies.

### Refinement

The amino H atom was found in a difference Fourier map and treated as riding with N—H = 0.86 Å, and with *U*_iso_(H) = 1.2*U*_eq_(N). The other H atoms were added at calculated positions and refined using a riding model, with C—H = 0.93 Å (or 0.96 Å for methyl H) and *U*_iso_(H) = 1.2*U*_eq_(C) or 1.5*U*_eq_(C_methyl_) .

### Crystal data

**Table:**

C_12_H_11_ClFN_3_	*F*(000) = 520
*M**_r_* = 251.69	*D*_x_ = 1.399 Mg m^−^^3^
Monoclinic, *P*2_1_/*c*	Mo *K*α radiation, λ = 0.71073 Å
Hall symbol: -P 2ybc	Cell parameters from 1870 reflections
*a* = 12.0593 (7) Å	θ = 3.0–29.1°
*b* = 8.3684 (4) Å	µ = 0.31 mm^−^^1^
*c* = 12.8611 (6) Å	*T* = 293 K
β = 113.021 (6)°	Prismatic, colourless
*V* = 1194.54 (11) Å^3^	0.5 × 0.3 × 0.2 mm
*Z* = 4	

### Data collection

**Table:**

Enraf–Nonius CAD-4 diffractometer	*R*_int_ = 0.014
Radiation source: fine-focus sealed tube	θ_max_ = 25.1°, θ_min_ = 3.0°
Graphite monochromator	*h* = −7→14
phi and ω scans	*k* = −9→9
4692 measured reflections	*l* = −15→15
2125 independent reflections	3 standard reflections every 60 min
1750 reflections with *I* > 2σ(*I*)	intensity decay: none

### Refinement

**Table:**

Refinement on *F*^2^	Primary atom site location: structure-invariant direct methods
Least-squares matrix: full	Secondary atom site location: difference Fourier map
*R*[*F*^2^ > 2σ(*F*^2^)] = 0.034	Hydrogen site location: inferred from neighbouring sites
*wR*(*F*^2^) = 0.096	H-atom parameters constrained
*S* = 1.04	*w* = 1/[σ^2^(*F*_o_^2^) + (0.0466*P*)^2^ + 0.2577*P*] where *P* = (*F*_o_^2^ + 2*F*_c_^2^)/3
2125 reflections	(Δ/σ)_max_ = 0.002
156 parameters	Δρ_max_ = 0.17 e Å^−^^3^
0 restraints	Δρ_min_ = −0.21 e Å^−^^3^

### Special details

**Table:**

Geometry. All e.s.d.'s (except the e.s.d. in the dihedral angle between two l.s. planes) are estimated using the full covariance matrix. The cell e.s.d.'s are taken into account individually in the estimation of e.s.d.'s in distances, angles and torsion angles; correlations between e.s.d.'s in cell parameters are only used when they are defined by crystal symmetry. An approximate (isotropic) treatment of cell e.s.d.'s is used for estimating e.s.d.'s involving l.s. planes.
Refinement. Refinement of *F*^2^ against ALL reflections. The weighted *R*-factor *wR* and goodness of fit *S* are based on *F*^2^, conventional *R*-factors *R* are based on *F*, with *F* set to zero for negative *F*^2^. The threshold expression of *F*^2^ > σ(*F*^2^) is used only for calculating *R*-factors(gt) *etc*. and is not relevant to the choice of reflections for refinement. *R*-factors based on *F*^2^ are statistically about twice as large as those based on *F*, and *R*- factors based on ALL data will be even larger.

### Fractional atomic coordinates and isotropic or equivalent isotropic displacement parameters (Å^2^)

**Table:**

	*x*	*y*	*z*	*U*_iso_*/*U*_eq_	
Cl1	0.75856 (5)	−0.06428 (7)	1.24493 (4)	0.0666 (2)	
N2	0.57335 (12)	0.12020 (18)	1.15433 (11)	0.0470 (4)	
N3	0.70666 (11)	0.09036 (16)	1.05829 (11)	0.0407 (3)	
F1	0.46632 (9)	0.35255 (15)	0.89928 (9)	0.0694 (3)	
C1	0.66841 (15)	0.0648 (2)	1.14005 (14)	0.0426 (4)	
C4	0.63837 (14)	0.1897 (2)	0.97700 (13)	0.0401 (4)	
C3	0.53397 (14)	0.2537 (2)	0.98347 (14)	0.0448 (4)	
N1	0.66996 (12)	0.22514 (18)	0.89014 (11)	0.0484 (4)	
H1	0.6236	0.2882	0.8387	0.058*	
C2	0.50232 (14)	0.2189 (2)	1.07122 (15)	0.0460 (4)	
C6	0.77521 (15)	0.1655 (2)	0.87727 (13)	0.0445 (4)	
C11	0.7589 (2)	0.0547 (2)	0.79274 (17)	0.0627 (5)	
H11	0.6821	0.0171	0.7491	0.075*	
C5	0.39149 (18)	0.2840 (3)	1.08108 (19)	0.0684 (6)	
H5A	0.3501	0.3528	1.0180	0.103*	
H5B	0.4136	0.3437	1.1500	0.103*	
H5C	0.3395	0.1974	1.0816	0.103*	
C10	0.8577 (3)	0.0005 (3)	0.7736 (2)	0.0862 (8)	
H10	0.8482	−0.0745	0.7174	0.103*	
C8	0.98515 (19)	0.1680 (4)	0.9222 (2)	0.0824 (8)	
H8	1.0622	0.2052	0.9653	0.099*	
C9	0.9696 (3)	0.0584 (4)	0.8384 (3)	0.0925 (9)	
H9	1.0361	0.0229	0.8254	0.111*	
C7	0.88790 (16)	0.2248 (2)	0.94391 (16)	0.0560 (5)	
C12	0.9052 (2)	0.3424 (3)	1.03578 (19)	0.0804 (7)	
H12A	0.8506	0.4302	1.0065	0.121*	
H12B	0.9866	0.3812	1.0645	0.121*	
H12C	0.8896	0.2916	1.0956	0.121*	

### Atomic displacement parameters (Å^2^)

**Table:**

	*U*^11^	*U*^22^	*U*^33^	*U*^12^	*U*^13^	*U*^23^
Cl1	0.0636 (3)	0.0849 (4)	0.0599 (3)	0.0128 (3)	0.0333 (3)	0.0278 (3)
N2	0.0441 (8)	0.0583 (9)	0.0462 (8)	−0.0052 (7)	0.0260 (7)	−0.0057 (7)
N3	0.0368 (7)	0.0512 (8)	0.0382 (7)	−0.0006 (6)	0.0192 (6)	0.0011 (6)
F1	0.0534 (6)	0.0882 (8)	0.0641 (7)	0.0227 (6)	0.0204 (5)	0.0185 (6)
C1	0.0413 (9)	0.0500 (10)	0.0411 (9)	−0.0061 (8)	0.0213 (7)	−0.0017 (7)
C4	0.0362 (8)	0.0502 (10)	0.0348 (8)	−0.0044 (7)	0.0150 (7)	−0.0043 (7)
C3	0.0371 (9)	0.0516 (10)	0.0426 (9)	0.0027 (8)	0.0122 (7)	−0.0005 (8)
N1	0.0428 (8)	0.0680 (10)	0.0374 (7)	0.0082 (7)	0.0188 (6)	0.0104 (7)
C2	0.0382 (9)	0.0524 (10)	0.0514 (10)	−0.0051 (8)	0.0221 (8)	−0.0125 (8)
C6	0.0463 (9)	0.0549 (10)	0.0391 (8)	0.0032 (8)	0.0240 (7)	0.0082 (8)
C11	0.0787 (14)	0.0675 (13)	0.0503 (11)	−0.0011 (11)	0.0344 (10)	0.0004 (9)
C5	0.0537 (11)	0.0799 (14)	0.0853 (15)	0.0077 (10)	0.0419 (11)	−0.0074 (12)
C10	0.133 (2)	0.0771 (16)	0.0817 (16)	0.0254 (17)	0.0782 (18)	0.0111 (13)
C8	0.0516 (12)	0.118 (2)	0.0884 (16)	0.0097 (13)	0.0386 (12)	0.0359 (16)
C9	0.095 (2)	0.110 (2)	0.109 (2)	0.0400 (17)	0.0793 (18)	0.0411 (18)
C7	0.0487 (10)	0.0710 (13)	0.0524 (10)	−0.0027 (9)	0.0240 (9)	0.0115 (9)
C12	0.0692 (14)	0.0959 (17)	0.0673 (14)	−0.0293 (13)	0.0171 (11)	−0.0094 (13)

### Geometric parameters (Å, º)

**Table:**

Cl1—C1	1.7384 (17)	C11—H11	0.9300
N2—C1	1.314 (2)	C5—H5A	0.9600
N2—C2	1.357 (2)	C5—H5B	0.9600
N3—C1	1.321 (2)	C5—H5C	0.9600
N3—C4	1.337 (2)	C10—C9	1.367 (4)
F1—C3	1.3533 (19)	C10—H10	0.9300
C4—N1	1.347 (2)	C8—C9	1.371 (4)
C4—C3	1.400 (2)	C8—C7	1.391 (3)
C3—C2	1.357 (2)	C8—H8	0.9300
N1—C6	1.433 (2)	C9—H9	0.9300
N1—H1	0.8600	C7—C12	1.488 (3)
C2—C5	1.494 (2)	C12—H12A	0.9600
C6—C11	1.383 (3)	C12—H12B	0.9600
C6—C7	1.385 (2)	C12—H12C	0.9600
C11—C10	1.384 (3)		
			
C1—N2—C2	114.88 (14)	C2—C5—H5B	109.5
C1—N3—C4	115.01 (14)	H5A—C5—H5B	109.5
N2—C1—N3	130.53 (16)	C2—C5—H5C	109.5
N2—C1—Cl1	115.19 (12)	H5A—C5—H5C	109.5
N3—C1—Cl1	114.28 (12)	H5B—C5—H5C	109.5
N3—C4—N1	119.86 (14)	C9—C10—C11	119.3 (2)
N3—C4—C3	118.98 (14)	C9—C10—H10	120.4
N1—C4—C3	121.15 (15)	C11—C10—H10	120.4
F1—C3—C2	121.27 (15)	C9—C8—C7	121.3 (2)
F1—C3—C4	117.46 (14)	C9—C8—H8	119.3
C2—C3—C4	121.27 (16)	C7—C8—H8	119.3
C4—N1—C6	124.73 (14)	C10—C9—C8	121.0 (2)
C4—N1—H1	117.6	C10—C9—H9	119.5
C6—N1—H1	117.6	C8—C9—H9	119.5
N2—C2—C3	119.31 (15)	C6—C7—C8	117.0 (2)
N2—C2—C5	117.72 (16)	C6—C7—C12	121.83 (17)
C3—C2—C5	122.97 (17)	C8—C7—C12	121.2 (2)
C11—C6—C7	122.05 (17)	C7—C12—H12A	109.5
C11—C6—N1	117.67 (16)	C7—C12—H12B	109.5
C7—C6—N1	120.19 (16)	H12A—C12—H12B	109.5
C6—C11—C10	119.4 (2)	C7—C12—H12C	109.5
C6—C11—H11	120.3	H12A—C12—H12C	109.5
C10—C11—H11	120.3	H12B—C12—H12C	109.5
C2—C5—H5A	109.5		
			
C2—N2—C1—N3	0.5 (3)	F1—C3—C2—C5	0.8 (3)
C2—N2—C1—Cl1	−178.98 (11)	C4—C3—C2—C5	−179.72 (17)
C4—N3—C1—N2	0.6 (3)	C4—N1—C6—C11	109.29 (19)
C4—N3—C1—Cl1	−179.90 (11)	C4—N1—C6—C7	−74.2 (2)
C1—N3—C4—N1	179.34 (15)	C7—C6—C11—C10	0.2 (3)
C1—N3—C4—C3	−1.3 (2)	N1—C6—C11—C10	176.61 (17)
N3—C4—C3—F1	−179.39 (14)	C6—C11—C10—C9	−0.5 (3)
N1—C4—C3—F1	−0.1 (2)	C11—C10—C9—C8	0.6 (4)
N3—C4—C3—C2	1.1 (2)	C7—C8—C9—C10	−0.4 (4)
N1—C4—C3—C2	−179.61 (16)	C11—C6—C7—C8	0.0 (3)
N3—C4—N1—C6	−0.9 (3)	N1—C6—C7—C8	−176.33 (16)
C3—C4—N1—C6	179.75 (16)	C11—C6—C7—C12	−179.19 (18)
C1—N2—C2—C3	−0.8 (2)	N1—C6—C7—C12	4.5 (3)
C1—N2—C2—C5	178.99 (16)	C9—C8—C7—C6	0.1 (3)
F1—C3—C2—N2	−179.44 (14)	C9—C8—C7—C12	179.3 (2)
C4—C3—C2—N2	0.1 (3)		

### Hydrogen-bond geometry (Å, º)

**Table:**

*D*—H···*A*	*D*—H	H···*A*	*D*···*A*	*D*—H···*A*
N1—H1···N2^i^	0.86	2.34	3.0768 (19)	145

Symmetry code: (i) *x*, −*y*+1/2, *z*−1/2.
